# Beta Blocker Use After Acute Myocardial Infarction in the Patient with Normal Systolic Function: When is it “Ok” to Discontinue?

**DOI:** 10.2174/157340312801215764

**Published:** 2012-02

**Authors:** Anna Kezerashvili, Kevin Marzo, Joshua De Leon

**Affiliations:** From the Department of Medicine, Cardiology Division, Winthrop University Hospital

**Keywords:** Acute myocardial infarction, Beta Blockers, Secondary prevention.

## Abstract

Beta-Blockers [BB] have been used extensively in the last 40 years after acute myocardial infarction [AMI] as part of therapy and in secondary prevention. The evidence for “routine” therapy with beta-blocker use post AMI rests largely on results of trials conducted over 25 years ago. However, there remains no clear recommendation regarding the appropriate duration of treatment with BBs in post AMI patients with normal left ventricular ejection fraction [LVEF] who are not experiencing angina, or who require BB for hypertension or dysrhythmia. Based on the latest ACC/AHA guidelines, BBs are recommended for early use in the setting of AMI, except in patients who are at low risk and then indefinitely as secondary prevention after AMI. This recommendation was downgraded to class IIa in low risk patients and the updated 2007 ACC/AHA guidelines suggest that the rationale for BB for secondary prevention is from limited data derived from extrapolations of chronic angina and heart failure trials. In this review, we examine the key trials that have shaped the current guidelines and recommendations. In addition, we attempt to answer the question of the duration of BB use in patients with preserved LVEF after acute MI, as well as which subgroups of patients benefits most from post AMI use of beta blockers.

## INTRODUCTION

The current guidelines on secondary prevention in patients status post acute myocardial infarction (AMI) recommend starting Beta Blockers (BB’s) for long term use (indefinite) in all patients (class I) [1]. These recommendations are based on several major trials including the Beta Blocker Heart Attack Trial (BHAT), the Norwegian Metoprolol Trial, Stockholm Metoprolol Trial, Cooperative Cardiovascular Project, Goteborg Trial, and a meta-analysis by Freemantle *et al*. [2-7]. The 2004 ACC/AHA guidelines changed this recommendation to class IIa in low risk patients [8], while the updated 2007 ACC/AHA guidelines believe that the rationale for BBs for secondary prevention is from limited data derived from extrapolations of chronic angina and heart failure trials [9-10]. The paucity of data may suggest the need to reevaluate recommendations regarding the duration of BB use in post AMI patients with normal left ventricular systolic function (LVSF). In this review, we examine the key trials that have shaped the current guidelines and recommendations. We review the immediate (days-months) and long term (years) effects of BBs on mortality and morbidity in patients after an AMI both in the pre and post-thrombolytic era. We will address the question: on which subgroups of patients, or upon which complications of acute myocardial infarction do BBs confer the most benefit? In addition, we will attempt to answer the question of the appropriate duration of BB use in patients with preserved LVSF after acute MI. 

Beta-Blockers have been used extensively in the last 40 years after (AMI) as part of primary therapy and in secondary prevention. They are employed for multiple indications such as hypertension [11], perioperative cardioprotection [12-13], angina [14] post cardiac surgery atrial fibrillation prevention [15-16] and arrhythmias. Early BB therapy has been recommended as part of the emergency treatment of suspected AMI, especially if the patient is tachycardic or hypertensive. Current recommendations for the use of BB’s in AMI are found in the 2004 Task force and 2004/2007 ACC/AHA STEMI guidelines. The evidence for “routine” therapy with beta-blockers post AMI rests largely on results of the trials conducted over 25 years ago. 

Despite these recommendations, there seems to be no clear consensus among cardiologists regarding the appropriate duration of treatment with BBs in post AMI patients with normal LVEF who are not experiencing angina, or who require BB for hypertension or dysrhythmia. Knowledge of the appropriate duration of treatment is relevant because of adverse effects of BB, including bradycardia, hypotension, bronchospasm, fatigue, reduced libido, depression, new onset diabetes and the additional medication burden.

Beta blockers reduce myocardial workload, and thus oxygen demand, via a reduction in heart rate and blood pressure [17-18]. They reduce catecholamine levels [19], decrease myocardial ischemia and limit infarct size, and may prevent the development of definite infarction in acute coronary syndrome (ACS) patients [18-25]. Early use of BBs in AMI has been shown to reduce the incidence of supraventricular and malignant ventricular arrhythmias, reduce the use of other anti-arrhythmic medications [7, 26-30], decrease chest pain symptoms [31], and decrease sudden cardiac death and early and late re-infarction [2, 7, 26, 28, 32-36]. 

Current recommendations regarding the use of BBs in the management of AMI as found in the ACC/AHA guidelines are highlighted here: BBs are currently recommended as long term-treatment for chronic, stable ischemic heart disease to control ischemia, prevent infarction and improve survival [1]. BBs are recommended to be used indefinitely in patients with decreased LVEF after AMI [9] and in patients with chronic heart failure in New York Heart Association (NYHA) functional class II-IV [37-40]. They are recommended for early use in the setting of AMI, except in patients who are at low risk (normal or near-normal LVEF, who have been successfully reperfused, or in the absence of significant ventricular arrhythmias). Listed contraindications include severe LV dysfunction. These recommendations are based on several large trials including ISIS-1, MIAMI, TIMI-IIb and GUSTO-I, all of which evaluated the effect of beta blockers during the acute phase of MI [34-35, 41-42]. The benefits of routine early intravenous (IV) use are felt to be less impressive based on the data obtained in the reperfusion era.

## SHORT TERM BENEFIT IN THE PRETHROMBOLYTIC ERA

The Goteborg trial [7] was one of the first randomized, double-blinded trials to demonstrate the beneficial effect of BBs on survival during the early phase of AMI. The protocol randomized 1,395 patients to metoprolol vs. placebo. Intravenous metoprolol was initially given followed by oral metoprolol. The authors found that patients treated within 12 hours of onset of ischemic pain had lower LDH values, (a measure of infarct size) and a 16% decrease in index infarctions**. **In the subsequent 90 days, there was a 36% reduction in mortality, 35% reduction in late MI and a decrease in ventricular fibrillation (VF) in the metoprolol-treated group. The reduction in mortality was similar in all defined subgroups. All patients were placed on open treatment with metoprolol after 90 days of double-blinded therapy. The mortality difference between the two groups was maintained at 1 year. The authors recommended the use of metoprolol during the early phase of AMI followed by long-term treatment, without specifying the actual duration. They only report one year follow-up, so benefit beyond that is only speculative.

The MIAMI trial (Metoprolol in acute myocardial infarction) [35] was the next major randomized double-blind placebo-controlled trial designed to test the benefit of BBs in suspected AMI. It randomized 5,778 patients to IV metoprolol or placebo within 24h of symptom onset, followed by oral treatment for 15 days. Overall there was no statistical difference in mortality between the treatment and placebo groups. In a retrospective analysis, the “high risk” patients demonstrated a 29% decrease in mortality rate. The authors suggested interpreting the P-value of 0.033 with caution as this analysis was made retrospectively. There was a non-significant decrease in the number of episodes of VF and re-infarction. The authors did report a significant decrease in development of definite infarction and reduction in tachyarrythmias with metoprolol therapy. The beneficial effect of metoprolol was limited to patients treated within 7 hours of symptom onset. The authors noted that the overall mortality rate was lower than predicted, and concluded that early institution of metoprolol did not reduce mortality. They suggested that this was due to selection bias. 

A comprehensive analysis by Yusuf *et al*. [23] followed the MIAMI and Goteborg trials and examined mortality and morbidity rates in randomized trials of beta-blockers after AMI. They found that short term treatment demonstrated a slight, non-significant benefit in mortality (3.4% vs. 3.6%, treatment vs. control), and no effect on ventricular arrhythmias or infarct size. The analysis suggested that because many trials used only oral BB, or did not initiate therapy until up to 72 hours after onset of chest pain, episodes of ventricular arrhythmias, which commonly occur in the early hours of AMI, may have been “missed”. The study did show significant reductions in cardiac enzyme release, chest pain and development of full MI in “threatened infarction” in patients treated within the first few hours of the onset of pain. It is important to note that these reductions were seen in only 12 of the 27 trials reviewed. 

The First International Study of Infarct Survival-ISIS-1 [34] randomized 16,027 patients to atenolol given IV immediately, followed by oral administration for 7 days. The study showed a 15% relative risk reduction in vascular mortality during the treatment period, but benefit was limited to administration during days 0-1. The study also showed significantly lower overall vascular mortality at one year, assumed to be due to the more likely use of BBs at discharge in the atenolol group [35% vs. 25% in controls]. The benefit was lost with follow-up beyond one year. Treatment also resulted in a significant increase in use of inotropic agents, non-significant increases in complete heart block, non-fatal re-infarctions and cardiac arrests, and no effect on the magnitude of cardiac enzyme release. The study did suggest that early IV treatment might produce ~15% reduction in the odds of cardiac arrest and an 18% reduction in the odds of early re-infarction. The authors concluded that the early gains of net decrease in death, cardiac arrest and re-infarction would persist after day 7 of treatment. They recommended to view these findings with caution, because they believed that BBs were prescribed for lower-risk patients. The ISIS-I study was not able to confirm the findings of the MIAMI trial, where benefit was seen retrospectively in “high risk” patients. The ISIS authors suggest that the findings “might be generalizable” but not to those at high risk [35]. 

## BETA BLOCKER USE WITH THROMBOLYTIC THERAPY

Trials addressing the use of early IV beta blockade were conducted after the widespread use of reperfusion therapy began. The first major study of AMI in the thrombolytic era which also addressed early use of BBs was the Thrombolysis in Myocardial Infarction (TIMI II-B) study [42]. It assessed the effects of immediate versus deferred BB therapy in patients receiving IV recombinant tissue-type plasminogen activator. The hypothesis tested was based on the rationale that with thrombolytic treatment, the highly vulnerable period for SCD may start earlier than the conventional period of 6-12 weeks in the non-thrombolytic experience. The study randomized 1,434 patients to immediate IV metoprolol followed by oral administration, or to deferred therapy where oral administration began on day 6. The authors found that immediate beta-blockade produced no improvement in LVEF, nor reduced mortality (in both invasive and non-invasive treatment arms) at hospital discharge. The authors believed that BB treatment did not change LVEF sufficiently to be detected (the primary end point) since patients with depressed LVEF were excluded. In fact, because of numerous exclusion criteria, the randomized population was a low risk group. In the secondary endpoints analysis, the study showed that early administration of BBs resulted in a lower incidence of re-infarction (2.7% vs. 5.1%, p=0.02) and recurrent chest pain (18.8% vs. 24.1%, p<0.02) at 6 days versus deferred therapy, but no difference at one year (8.6% vs. 9.6%, p=0.44) In conclusion, immediate BB use was recommended for prevention of ischemia and re-infarction during the first week following thrombolytic therapy, but offered no benefit over late administration. Mean discharge LVEF in both groups was 50% and was unchanged at one year. Again, later follow-up was not reported, and in the absence of a non BB-treated cohort, benefit beyond one year remains at best speculative. 

Pfisterer and colleagues, in the pre-specified post-hoc analysis of the GUSTO-I trial, reported the results of a non-randomized observational study, examining 30-day mortality of fibrinolytic-treated patients with ST-elevation MI who received any atenolol (IV, oral or both) or no atenolol [41] Use of any atenolol conferred a 5-fold lower mortality risk than if no atenolol had been given, but the majority of the benefit was limited to oral use only. Overall, use of atenolol in GUSTO-I was associated with decreased mortality, stroke, shock and arrhythmias, but increased recurrent ischemia and re-infarction. The authors reported that interpretation of this data is confounded by the fact that atenolol-treated patients were less ill at presentation and may have already had preservation of LV function. The authors concluded that IV atenolol adds only limited value to early oral atenolol and recommended to begin oral atenolol once the patient is clinically stable. 

A meta-analysis by Freemantle *et al* published in 1999 reported on 82 randomized trials which examined the effects of BBs on all cause mortality with both short and long term treatment [6]. Review of the 51 short term trials involving 29,260 patients showed a small and non-significant reduction in the odds of death (0.4 deaths in 100 patients). The authors concluded that short term beta blockade immediately after myocardial infarction is unlikely to be of major benefit unless treatment is continued long term.

The Commit study, one of the largest trials, randomized 45,852 patients to early IV metoprolol followed by oral metoprolol or placebo within 24 hours of suspected AMI onset. Treatment was up to discharge or 4 weeks in the hospital [43]. This study included Killip class II and III patients (24%), who were excluded in many of the previous trials [35]. Approximately 50% of the patients in this study received fibrinolytic therapy, and patients scheduled for primary percutaneous coronary intervention (PCI) were excluded. The study showed that early BB therapy had no effect on the primary composite outcome of death, re-infarction or cardiac arrest, and no effect on the co-primary outcome of death alone. Treatment did reduce the risks of re-infarction by 18%, ventricular fibrillation by 17% and death due to arrhythmia by 22%. These reductions emerged gradually, beginning on day 2, but were counterbalanced by a 29% increase in death due to cardiogenic shock and a 12% increase in development of CHF. The authors believed this increase in shock was seen in high risk patients, and there was a tendency toward a net benefit in low-risk patients. They proposed that early after the onset of MI, higher-risk patients may be poorly perfused, and rapid reduction in blood pressure with beta-blocker therapy may further compromise the patients’ hemodynamics [44]. It was concluded that BB therapy be started only after a patient’s hemodynamic condition has stabilized. 

Al-Reesi *et al.* (2008) revisited the data on the use of BBs in acute MI in a meta-analysis of 18 studies with a total 74,643 patients [45]. This review examined randomized controlled trials assessing 6-week mortality in patients receiving BB within 72 hours of acute MI. Fifteen out of the eighteen trials excluded patients with CHF. The study found no survival benefit from acute intervention with BB at 6 weeks, while a subgroup analysis after exclusion of high-risk patients (Killip class III and IV) showed a small but significant absolute risk reduction of 0.4% in short-term mortality [45]. The explanation offered for the lack of cardiovascular protection in the early phase post MI was that the myocardium might be stunned immediately after AMI, resulting in depressed ejection fraction, and the use of beta-blockers may worsen myocardial contractility under these conditions. 

Review of the data regarding the benefits of short term use of BBs for reduction of mortality in AMI appears to be equivocal. Even when patients are segregated by risk category, the data is conflicting. Some trials (TIMI-IIB, ISIS, PAMI) [34, 42, 46] showed mortality reduction in low risk groups, while MIAMI [35] and GUSTO-I [41] showed reduction only in high risk groups. The Goteborg trial showed reduction in mortality in all groups[7], and the Yusuf at al meta-analysis showed only a slight, non-significant benefit in mortality with BB therapy [23]. The data on reducing re-infarction and tachyarrhythmias seems to favor early BB use, as confirmed in most trials completed during both the pre-fibrinolytic and fibrinolytic eras [7, 34-35, 42-43]. Thus, early oral BB therapy may reduce short term overall mortality (0-6 weeks) in both low and high risk patients, as well as reduce the rates of re-infarction and tachyarrythmias, but the data remains inconclusive. The data does suggest that the early use of IV BBs may result in higher risk of cardiogenic shock and death.

## IS THERE A LONG TERM BENEFIT OF BETA-BLOCKER USE?

One of the earliest studies suggesting benefit of beta-blockers after AMI was the Norwegian Timolol Trial [2]. It was a double-blind randomized study of 1,884 patients which examined the effect of timolol administered 7-28 days after AMI, and followed patients for 12-33 months. The study was conducted primarily in low risk, “clinically stable” patients. The study employed an “intention-to-treat” analysis. The authors found that use of timolol resulted in a 39.4% reduction in mortality, a 44.6 % reduction in sudden-death, and a 28.4% reduction in re-infarction rates. The mortality curves continued to diverge up to 30 months, after which the curves became parallel. The authors report that after 24 months the number of at risk patients was too small to derive conclusions with respect to mortality, and report a negligible difference between the two curves for re-infarction after six months. These findings were similar to those in the American beta-blocker Heart Attack Trial (BHAT) [47], the Goteborg Metoprolol Trial [7] and the Stockholm Metoprolol Trial [4]. 

Pedersen *et al* reported 6 year follow-up of the Norwegian Timolol Trial patients with severe angina, hypertension or cardiac arrhythmias treated with BBs for longer than 36 months (vs. placebo-treated patients for the same period) [48]. The benefit obtained during the first year was preserved and the curves continued to diverge until 72 months of follow-up. The benefit was seen in the older and high risk patients only. There was no significant difference at 72 months in low-risk patients [49].

The BHAT trial followed the Norwegian Trial and was halted early due to a significant mortality reduction with BB treatment. The BHAT trial randomized 3,837 patients 5-21 days after acute MI to propranolol, a non-selective BB, or placebo with a mean follow-up of 27 months. Patients with bradycardia, CHF and prior BB treatment were excluded. Total mortality was reduced by 25%, sudden death by 28% and arteriosclerotic heart disease by 27%. The survival curves diverged for the first year, then became parallel. The benefits of propranolol were found to be similar in all risk groups. The authors note the limitation that the study was not designed to answer the question of duration of treatment with BBs after AMI, but based on the finding that the beneficial effects were most pronounced at 12-18 months, and sustained up to 39 months, the investigators recommended the use propranolol for at least 3 years. 

A sub-analysis of the BHAT database by Viscoli *et al* assessed the mortality rates in the study population after division into low, medium and high risk groups, and by 12 months or more of treatment [50] . The authors found that while propranolol therapy conferred an improvement in mortality of 43% among high risk patients, there was no evidence of long term benefit in the low risk population, calculated to be approximately 88% of the cohort. In addition, it highlighted that the overall risk reduction of 27% seen at 2 years occurred primarily in the first year, during which the risk reduction was 39%. In fact the risk reduction declined to 18% after 1 year, once adjusted for the effects of differences in the risk for death [50]. Overall, the authors concluded that the benefits of propranolol treatment in BHAT were confined to the highest risk patients. They also suggested that physicians and patients with an uncomplicated course after MI may want to reconsider the continued use of beta blockers beyond 1 year.

The findings of BHAT were further evaluated in a post-hoc analysis by Georghiade *et al*, who examined the cohorts with either Q-wave or non-Q-wave MI’s, the latter representing 17% of the BHAT population. This analysis found the cumulative mortality in the propranolol and placebo-treated non-Q wave MI groups to be 7.8 and 7.9% respectively (non-significantly different). In the Q-wave MI cohort there was a statistically significant 27% relative risk reduction in mortality compared with placebo. The authors concluded that the beneficial effects of propranolol were limited to the Q-wave MI population [51]. 

Another analysis of BHAT by Hawkins *et al*, found propranolol to improve mortality in older patients [3]. The study examined patients aged 30-59 years vs. 60-69 years. The older group had a 33.3% reduction in mortality versus placebo, compared to an 18.9% reduction in the younger age group. In this latter group, benefit from propranolol was confined to the first 6 months, while in the older group there was a continuing separation of the curves up to 36 months. 

The Stockholm Metoprolol study published in 1985 [4] was a double-blind study which randomized 301 patients post MI to oral metoprolol vs. placebo and followed them up to 36 months. Patients with prior need for beta-blockade or patients in heart failure, atrial fibrillation, or with obstructive pulmonary disease or hypotension were excluded. There was a significant reduction in cardiac death in patients with a large infarct (32% vs. 12.5%), and a significant decrease in sudden death (59%) and nonfatal re-infarction rates (45%) in the metoprolol group. The curves for both sudden death and total mortality continued to diverge throughout the follow-up period. The total mortality between the two groups was not significantly different. The authors reported that the significant reduction in cardiac mortality was in patients with a large infarct or in patients over 64 years of age. This difference was mainly driven by reduction in sudden deaths. The authors concluded that therapy should be continued for at least 3 years. They do not mention the use of aspirin or ejection fraction, so applicability of this data is limited.

Yusuf *et al*’s review of 16 randomized trials on long term (1-3 years) prophylactic use of beta blockers after MI reported that a “crude overview” of the results suggested a moderate reduction of the absolute risk of death from 10% to about 8% [23]. The analysis also showed a 25% reduction in odds of re-infarction, and a 30% reduction in the odds of sudden death. The authors state that the trials reviewed were too small to identify subgroups of patients for whom treatment was advantageous. Very few studies in the review showed significant decreases in re-infarction rate, but when the data was pooled, there was a significant difference between the control and treatment groups. Yusuf *et al* believed that the analyses of sudden death to be unreliable, since the definition of sudden death varied from trial to trial. They conclude that only long term treatment trials demonstrated benefit in reducing mortality, which may be because more patients have been randomized to long term trials compared to short term trials as of the time of their review.

Another large meta-analysis by Olsson *et al* examined several long term double blind studies to determine if metoprolol reduced post-infarction mortality [52]. The five trials that were evaluated included the Goteborg Metoprolol trial and Stockholm Metoprolol trials described above, along with the Amsterdam Metoprolol Trial, Belfast Metoprolol trial and Lopressor Intervention Trial [53-54]. The pooled mortality rates were 19% lower in the metoprolol treated patients, compared with non-treated patients. Use of metoprolol showed a 40% reduction in sudden cardiac death. Mortality reduction was independent of gender, age and smoking habits, and was driven mainly by reduced sudden cardiac deaths. In review of the two figures representing the cumulative number of all deaths, it is evident that the two mortality curves separate early, with the major difference in mortality occurring during the first year. After one year, the curves became parallel. Only in the Stockholm Metoprolol trial did the curves for sudden death and cardiac mortality continue to diverge throughout the 36 months of follow-up [4]. 

The meta-analysis by Freemantle *et al* included 31 long term trials involving 24,974 patients, and the analysis concluded that there was a 23% reduction in the odds of death with Beta blocker use [6] The analysis looked at annual reduction in mortality across the trials. The findings suggested an annual reduction of 1.2 deaths and 0.9 re-infarctions for 100 patients treated. When looking at predictors of benefit, initiation with intravenous dose did not add additional benefit, but the authors found no reason to delay, as early initiation would lead to a greater period when benefit may be accrued from treatment. Use of BBs with intrinsic sympathomimetic activity showed a non-significant trend towards reduced benefit (OR 1.10). The meta-analysis concluded that there was no loss of benefit over time, as additional therapeutic options for treatment were introduced, particularly thrombolytic agents and aspirin. In their discussion, the authors highlighted that in their analysis, the number needed to treat to avoid one death was 42 with BBs compared to 153 and 94 with antiplatelet agents or statins, respectively. The authors suggested that beta blockers should be continued indefinitely. 

The APSI trial [55] which followed in 1997 attempted to explore the effect of long term treatment with BBs on mortality after acute MI in high risk patients. The study randomized 607 patients to 1 year of treatment with acebutolol or placebo, and had a median follow-up of 6 years. There was a 48% relative reduction in total mortality at 1 year. The difference in mortality was significant in the first year after which the survival curves remained parallel. Their analysis suggested that the initial benefit may remain until the fourth year. The patients’ treatments were specified and blinded only for the first year, after which there is no knowledge whether the patients were on BB or not. Thus, this study could not provide evidence about the optimal duration for beta-blocker therapy after AMI.

The trials reviewed above, both long term and short term were performed in the era before anti-platelet therapy and PCI had become routine, and the average mortality in the control population in these studies was approximately 4%, as the trials included primarily low-risk patients. In addition, these trials were representative of BB effects on patient populations under-treated by current standards. The patients in these trials were not treated as aggressively with ACE inhibitors, aspirin and statins as is recommended by current post-MI standards of care. Thus, it is difficult to assert that the albeit inconsistently positive effects of beta-blocker use found in these trials are definitively applicable in the current era of treatment.

One of the few studies to examine this issue of applicability was an analysis by Kernis *et al* of 2,442 patients who underwent successful primary PCI [56] published in 2004. The study examined the outcome of patients treated with or without BBs in the PAMI-2, PAMI No SOS, Stent PAMI and Air PAMI trials. In these trials, the percent of the patients given BBs post-PCI ranged from 53% (Air PAMI) to 82% (PAMI-No SOS). In multivariate analysis, beta-blockers were independently associated with lower six-month mortality and Major Adverse Cardiac Events (MACE), with the benefit seen in patients with decreased LVEF (<50%) and multi-vessel coronary artery disease. The beneficial effect was NOT seen in patients with LVEF>50% and single-vessel CAD. The Kernis *et al*. speculated that complete and successful primary revascularization in patients with normal LVEF reduce the risk of cardiac events to a point where chronic beta-blockade becomes unnecessary. They also suggested that the presence of a selection bias favoring use of beta-blockers in healthier patients may have led to improved prognosis in BB patients overall.

An analysis of the Cadillac trial by Halkin *et al*, extended the findings of Kernis *et al* (2004) by examining the effect of IV BB administered before PCI on survival after AMI [57] Pre-procedural BB were administered in 1,136 patients and withheld in the remaining 946 patients. The patients in the BB group were younger, had more hypertension and anterior infarction with depressed LVEF than patients not treated with pre-procedural beta-blocker. The 30-day mortality was significantly lower in the BB group (1.5% vs. 2.8%), but only for patients without prior BB therapy. In addition, no reduction in mortality was observed after 1 year. There were no differences in the 30-day rates of re-infarction, target vessel revascularization, or stroke. The authors concluded that patients unprotected at the time of AMI onset by long-term BB therapy would derive the greatest clinical benefit from IV BB administration before PCI, but one could reasonably conclude that these findings are limited in applicability and may not support long term use.

## CONCLUSIONS

In summary, the Norwegian Timolol Trial, the BHAT and Stockholm trials [26-27, 33] all showed a reduction in mortality, sudden death, and re-infarction for up to 30-36 months, but these benefits were engendered by reductions occurring in the first year of therapy [47, 50]. The benefit was limited to high risk patients [2, 50], older patients [3-4, 47], and patients with large [4] or Q-wave infarction [51].The benefits persisted over 36 months in patients with severe angina, hypertension and arrhythmias only in the 6 year follow-up of the Norwegian trial [48]. The findings of the APSI trial suggested the persistence of benefit up to 4 years in high-risk patients, but the benefit developed in the first year without change over time [55]. Reduction in sudden cardiac death was significant in all trials. The Yusuf at al meta-analysis also confirmed a reduction in total mortality, re-infarction and sudden death with long term [1-3 year] BB use, without specifying which subgroup benefitted most. Olsson’s review confirmed the reduction in mortality and sudden cardiac death up to 3 years post myocardial infarction, but the curves of death and sudden death diverged significantly only during the first year, and become parallel afterwards. Only the meta-analysis by Freemantle, which showed an annual reduction of 1.2 deaths and 0.9 re-infarctions per 100 patients treated with BBs, provides evidence that the beneficial effect on mortality with BB treatment after AMI may continue to accrue after the first year. Kernis at el showed that beta-blockade in patients treated with PCI lowered six-month mortality and MACE, but only significantly in patients with depressed ejection fraction and multi-vessel CAD [56]. BB treatment with PCI produced a decrease in 1-year mortality, but this was driven by the decrease in in-hospital deaths [46]. 

It should be noted that the differences or similarities in findings across the trials does not appear to be related to differences in the beta-blocking agent used. The majority of the studies employed metoprolol or atenolol, beta-1 selective agents, and the type of long or short term benefit seen, if present at all, did segregate to one agent or the other. Trials which employed non-selective agents, including BHAT and the Norwegian Timolol Trial, demonstrated similar findings. The APSI trial, which studied acebutolol, a beta-1 selective agent with sympathomimetic activity, also demonstrated similar findings to studies with metoprolol or atenolol. The meta-analysis by Freemantle *et al* noted that trials employing agents with sympathomimetic activity showed a trend toward decreased benefit. Thus, variability of the findings does not appear to be explained by differences in the beta-blocker used. Studies of the use of agents such as carvedilol or bisoprolol in post-AMI patients show clear reductions in mortality, but these were demonstrated primarily in heart failure populations, with Left Ventricular systolic dysfunction. Therefore, these studies are not included in the present discussion.

This review of the literature demonstrates that the data upon which current guidelines for beta-blocker use post-AMI are based is conflicting, and is at best suggestive rather than directive. The majority of the data suggests that there is a reduction in total mortality, re-infarction and sudden cardiac death in the first 3 years of beta-blocker use, especially in patients who are at high risk, but the benefit emerges in the early phase after MI. Additional benefit does not appear to reliably accrue beyond one year. Overall, low-risk patients benefit the least from BB therapy, and thus it is reasonable to conclude that there is not strong support for continued treatment of patients with BB for more than one year post MI, particularly in low risk patients or those with preserved LVEF. 

In conclusion, it can be recommended that acutely, all hemodynamically stable AMI patients receive BB to reduce chest pain, as well as to reduce the risk of re-infarction and Ventricular arrhythmias. There may be a slight reduction in mortality if the patient has not previously been treated with BB. However, it remains inconclusive whether BB use beyond one year truly reduces mortality in the current era of AMI and post-AMI care, particularly in patients with preserved Left Ventricular systolic function. In these and other low risk patients (young, no arrhythmias or residual ischemia), prolonged use with Beta-Blockers is unlikely to confer mortality benefit. These findings can inform a practitioner’s decision regarding the risks and benefits of discontinuation of BBs in low risk patients, in the not infrequent clinical circumstance where discontinuation needs to be considered. It is important to keep in mind that most of the trials reviewed above were conducted before the widespread use of revascularization either by thrombolysis or PCI. Further studies are warranted to examine the effect of the duration of treatment with beta-blockers in asymptomatic patients treated with current medical therapy and interventions.

## ABBREVIATIONS

ACS: = Acute coronary syndrome
AM I: = Acute myocardial infarction
BHAT: = Beta blocker heart attack trial
BB: = Beta blockers
LVE F: = Left ventricular ejection fraction
LVSF: = Left ventricular systolic function
MIAMI: = Metoprolol in acute myocardial infarction
NYHA: = New york heart association
